# Recurrent, Severe Coital Headaches Associated With Bilateral Carotid Artery Aneurysms and the Effect of Endovascular Treatment

**DOI:** 10.7759/cureus.59289

**Published:** 2024-04-29

**Authors:** Emma L Martinez Arellano, Hannah Lu, George Ishac, Hashem Shaltoni, Ruiqing Sun

**Affiliations:** 1 Neurology, University of Texas Medical Branch, Galveston, USA

**Keywords:** migraine headaches, flow diverting stent, headache associated with sexual activity, carotid artery aneurysms, coital headache

## Abstract

Headaches are one of the most common chief complaints in the outpatient setting. Distinguishing between benign and life-threatening headaches can be difficult, particularly in the setting of a pre-existing history of headaches. Here, we present a 41-year-old female with a past medical history of migraines and uterine leiomyoma status post hysterectomy about nine months ago who presented to the clinic for severe coital headaches and worsening migraines starting eight months ago. Computer tomography angiogram (CTA) head and neck demonstrated bilateral para-ophthalmic internal carotid artery (ICA) aneurysms (right, 7.5, left 6 mm). A diagnostic cerebral angiogram (DSA) was subsequently done and confirmed the CTA findings. The patient underwent left and right flow-diverting stent placement two and four months later, respectively. One week after the right ICA stent placement, her headaches had improved to one to two times per week. At six months after the stent placement, she resumed her normal sex life and her migraines returned to baseline. Our case suggests that recurrent severe coital headaches are associated with bilateral carotid artery aneurysms. Thus, while assessing a patient with recurrent coital headaches, it is important to have a wide arsenal of differentials to rule out possibly catastrophic causes.

## Introduction

Headaches associated with sexual activity (HASA) are described as those that occur during sexual activity and are either abrupt in onset or intensifying with increasing sexual excitement [1]. Malignant coital headaches can present as acute emergencies involving hemorrhagic or ischemic events while others can present as insidious processes consisting of cerebral aneurysms, arteriovenous malformations, hydrocephalus, or reversible cerebral vasoconstriction. An estimated 3.8% to 14.5% of patients had an aneurysmal rupture resulting in subarachnoid hemorrhage immediately following sexual activity [2]. Thus, timely intervention and standardized diagnostic workup of associated comorbidities are instrumental in preventing fatalities. There are only two reported cases of recurrent coital headache related to an unruptured saccular aneurysm of the carotid artery and fusiform aneurysm of the vertebral artery respectively [3,4].

This case report was previously presented as a poster at the 2023 American Neurological Association meeting on September 10, 2023.

## Case presentation

**Figure 1:**
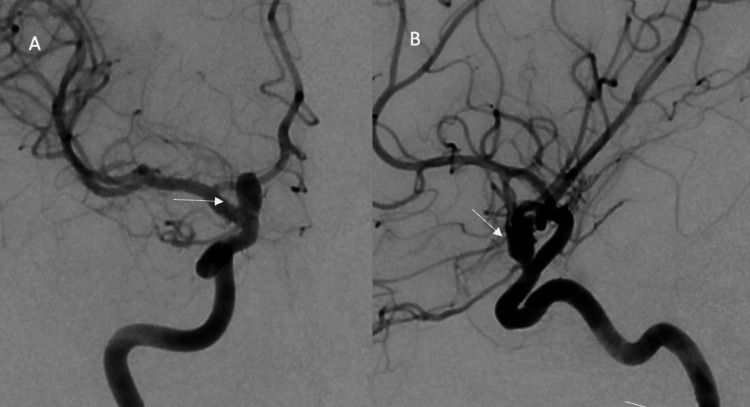
(A) Anterior view of the right internal carotid artery (ICA) aneurysm diagnostic cerebral angiogram (DSA); (B) Lateral view of left ICA aneurysm DSA

**Figure 2:**
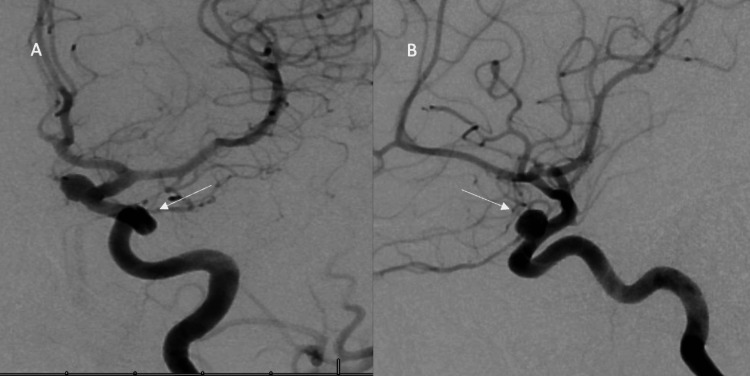
(A) Anterior view of left internal carotid artery (ICA) aneurysm diagnostic cerebral angiogram (DSA); (B) Lateral view of the left ICA aneurysm DSA

A 41-year-old female with a past medical history of migraines, blood loss anemia, and uterine leiomyoma status post hysterectomy nine months ago presented to the clinic for severe coital headaches starting eight months ago. Her coital headache was sudden in onset and explosive in nature with gradual resolution of symptoms in varying timeframes (30 minutes to 2 hours). Simultaneously, she began having frequent, severe migraines about three to four times weekly that were refractory to medical management. Her baseline was two episodes of migraine headaches per month. Neurological examination on initial presentation was unremarkable. Computer tomography angiogram (CTA) head and neck demonstrated bilateral para-ophthalmic internal carotid artery (ICA) saccular aneurysms (right, 7.5, left 6 mm). A diagnostic cerebral angiogram was subsequently done and confirmed the CTA findings (Figures 1A, 1B, 2A, 2B).

**Figure 3:**
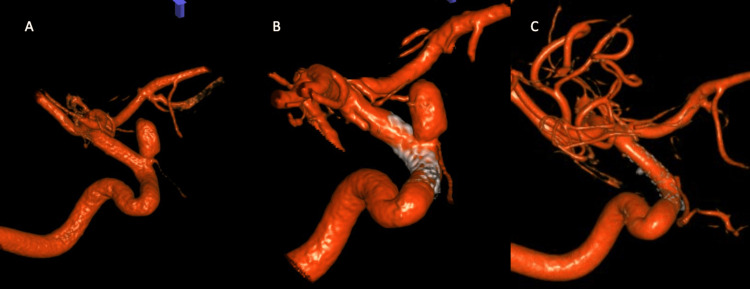
(A) 3D reconstruction of right internal carotid artery (ICA) aneurysm pre-treatment; (B) 3D reconstruction of right ICA immediately after flow-diverting stent placement; (C) 3D reconstruction of the right ICA six months after flow-diverting stent placement

**Figure 4:**
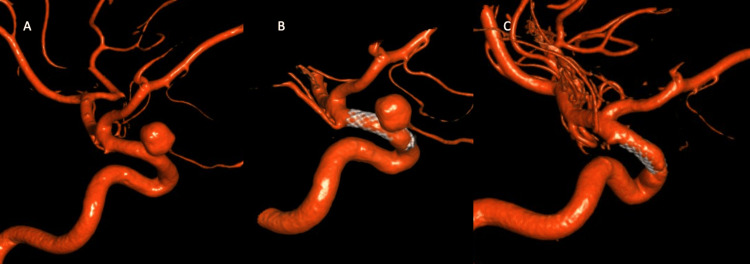
(A) 3D reconstruction of the left ICA aneurysm before intervention; (B) 3D reconstruction of the left ICA immediately status post flow-diverting stent; (C) 3D reconstruction of the left ICA six months after flow-diverting stent placement

The patient underwent left and right flow-diverting stent placement two and four months later, respectively (Figures 3B, 4B). One week after the right ICA stent placement, her headaches had improved to one to two times per week. Six months after stent placement, she resumed her normal sex life and her migraines returned to baseline. The patient underwent repeated cerebral angiogram after five months of a right ICA aneurysm intervention, which demonstrated complete obliteration of a right ICA aneurysm (Figure 3C) and left ICA aneurysm (Figure 4C) compared to pre-intervention (Figures 3A, 4A).

## Discussion

Ruptured intracranial aneurysms are by far the most common cause of non-traumatic subarachnoid hemorrhage, which is a neurological emergency with potentially devastating consequences. There is evidence that some of the cerebral aneurysm rupture cases are precipitated by sexual intercourse and intense physical exertion, which increase the risk of aneurysm rupture by as much as 15-fold [5]. Coitus and physical exercise share some physiological similarities - hyperdynamic alterations in blood pressure, heart rate, respiratory rate, and muscle tone, which might be potential precipitants for cerebral aneurysm growth and rupture. However, Reynolds et al. demonstrate that sexual intercourse has a higher risk for aneurysm rupture than physical exercise alone [2]. During sexual intercourse, there is an increase in the transmural pressure across a preexisting aneurysm wall by increasing the mean artery blood pressure (MABP) and/or decreasing the intracranial cerebral pressure. In contrast, during physical exercise alone, the increase of MABP is counterbalanced by the increase of intracranial pressure. Rapid stretching of the aneurysm wall during intercourse might be the reason for HASA.

Coitus was the immediately preceding activity in 3.8-14.5% of patients suffering from aneurysmal subarachnoid hemorrhage. Therefore, HASA may be an important harbinger of a cerebral aneurysm with the potential for future rupture. Early recognition and neurovascular imaging are warranted. When HASA first occurs, it is critical to exclude secondary causes of headache, which include cerebral aneurysm [4,5], subarachnoid hemorrhage [6], cerebral artery dissection [7,8], reversible cerebral vasoconstriction [9], and cerebral ischemic or hemorrhagic stroke [9-11]. Two existing case reports describe secondary sexual headaches associated with unruptured, unilateral aneurysms. HASA attributed to unruptured, bilateral, saccular aneurysms has yet to be reported in the literature. This case reports a patient with a longstanding history of migraine with new-onset HASA who was found to have bilateral ICA saccular aneurysms and was managed with flow-diverting stents successfully.

In our case, the endovascular intervention (bilateral flow diverting stent placement) not only resolved the coital headache but also improved the patient’s regular migraine. This is consistent with previous studies that demonstrated coil embolization of unruptured aneurysms resulted in a reduction of the severity of regular headaches in a majority of patients [12-14].

There is a high percentage of subarachnoid hemorrhage resulting from aneurysmal rupture immediately following sexual activity [2]; however, there are few case reports regarding unruptured cerebral aneurysm associated with coital headache, which raises the hypothesis that coital headache associated with an unruptured cerebral aneurysm is under-reported or under-diagnosed. Considering the good outcome of surgical intervention of cerebral aneurysms before a devastating hemorrhage occurs, it is evident that early diagnosis of intracerebral aneurysm associated with HASA is crucial.

## Conclusions

We report a case of recurrent severe coital headaches associated with bilateral carotid artery aneurysm diagnosed by CTA and diagnostic cerebral angiogram. The patient was successfully treated with bilateral flow-diverting stent placement resulting in improvement of headaches and quality of life. When evaluating a patient with coital headaches, it is important to keep a wide arsenal of differentials in order to rule out potential catastrophes such as aneurysms. Timely diagnosis and intervention with endovascular flow diversion or coiling can prevent fatalities and improve outcomes.
